# Data from monogenean and endohelminth communities in twospot livebearer *Pseudoxiphophorus bimaculatus* (Teleostei: Poeciliidae) populations in a neotropical river

**DOI:** 10.1016/j.dib.2020.106180

**Published:** 2020-08-16

**Authors:** Guillermo Salgado-Maldonado, Juan Manuel Caspeta-Mandujano, Edgar F. Mendoza-Franco, Miguel Rubio-Godoy, Adriana García-Vázquez, Norman Mercado-Silva, Ismael Guzmán-Valdivieso, Wilfredo Matamoros

**Affiliations:** a Instituto de Biología, Laboratorio de Helmintología, Universidad Nacional Autónoma de México, Apartado Postal 70-153, CP 04510, Ciudad de México, Mexico; b Facultad de Ciencias Biológicas, Laboratorio de Parasitología de Animales Silvestres, Universidad Autónoma del Estado de Morelos, Cuernavaca, Morelos, Mexico; c Instituto de Ecología, Pesquerías y Oceanografía del Golfo de México (EPOMEX), Universidad Autónoma de Campeche, San Francisco de Campeche, Campeche, Mexico; d Instituto de Ecología, A. C., km 2.5 Antigua Carretera a Coatepec, 91070, Xalapa, Veracruz, México; e Centro de Investigación en Biodiversidad y Conservación, Universidad Autónoma del Estado de Morelos, Cuernavaca, Morelos, Mexico; f Instituto de Ciencias Biológicas, Universidad de Ciencias y Artes de Chiapas, Tuxtla Gutiérrez, Chiapas, Mexico

**Keywords:** Platyhelminth, Monogeneans, Gyrodactylids, Trematodes, Nematodes, Parasites of freshwater fish, Abundance, Geographical distribution, Altitudinal gradient

## Abstract

The data presented in this article are related to the research article entitled “Competition from sea to mountain: interactions and aggregation in low diversity monogenean and endohelminth communities in twospot livebearer *Pseudoxiphophorus bimaculatus* (Teleostei: Poeciliidae) populations in a neotropical river.” accepted for publication in Ecology and Evolution. The data describes the communities of helminth parasites in 11 populations of a small poeciliid freshwater fish *Pseudoxiphophorus bimaculatus* (Heckel, 1848) sampled along the La Antigua river basin in Veracruz, Mexico. We examined 19 *P bimaculatus* from one locality, 21 from another locality, and 20 from each of the other nine locations sampled in June 2016. A total of 220 individual fish were examined, and in this paper we provide the data for 18 helminth parasite taxa recorded from them. The material in this Data paper comprised the raw data on the abundance, i.e. the number of helminth individuals of each of 18 taxa found in each one individual of *P. bimaculatus* from each of 11 localities. The data set is contained in a single text-table including one matrix containing each of the 220 host *P. bimaculatus* examined from 11 localities (lines). Measures for each host *P. bimaculatus* include total length, standard length, maximum deep and sex, documented for everyone fish examined, plus data of the number of individual helminth of each taxa collected by each examined fish are placed in the columns. These data might be used to examine spatial distribution of helminth parasite taxa. These data might be reused to examine the spatial variation in community structure of helminth parasites of freshwater fish. This kind of data could be used to provide an assessment of human environmental impacts, or for public awareness of conservation objectives.

**Specifications Table**

**Table:** 

**Subject**	*Biology; Animal science and Zoology*
**Specific subject area**	*Parasites of freshwater fish; Platyhelminth and Nematodes. Helminth ecto- and endo-parasites of tropical freshwater fish of Mexico.*
**Type of data**	*Text-Table.*
**How data were acquired**	*Microscope, survey. Each fish was examined under a stereo microscope in Petri dishes with river water for external examination, and with saline solution for internal organs. External examination included the skin, scales, mouth, gill cavity, anus, and fins of each host; while internal examination included the brain, gut, mesenteries, kidneys, liver, gall bladder and muscles. We collected data on the number of species (species richness) and abundance distribution of helminths (number of individuals of each species).*
**Data format**	*Raw numbers in matrix; text-table of fish-individuals in localities (lines)* vs *characteristics (location coordinates and altitude; host length, weigh and sex), and helminth taxa (columns) recorded in each one fish host individual. Single matrix containing each one of 220 fish individuals examined.*
**Parameters for data collection**	*We examined 19 P bimaculatus from Agua Bendita, 21 from Puente Nacional, and 20 from each of the other nine locations sampled in June 2016. Specimens were collected under collecting permit FAUT-0105. Fish were collected using DC backpack electroshockers, seines, and gill nets. Captured individuals were placed in plastic bags filled with water, transferred to the laboratory, and kept alive in aerated containers until subsequent examination for the presence of helminth parasites (within 8* *h of capture). To complete the examination, fish were euthanised with an overdose of the anaesthetic 2-phenoxyethanol (Sigma-Aldrich, St. Louis, Missouri), measured (total and standard lengths), and examined under a stereomicroscope in Petri dishes containing river water. Externally, the skin, scales, mouth, branchial cavity, anus, and fins of each host were examined. The branchial arches were removed, separated from the branchial cavity, and evaluated individually. All internal tissues, including the digestive tract, body musculature, and organs were examined for helminth parasites in Petri dishes containing saline 0.7% solution. The helminths that were obtained from the dissections were counted and recorded separately for each fish.*
**Description of data collection**	*All helminths (except for Gyrodactylid monogeneans) found were fixed in 4% hot formaldehyde, stained with Mayer's paracarmine or Gomori´s triple stain and mounted whole on Canada balsam, to get permanent slides for microscopical examination. Taxonomic identification of helminths was performed based on morphometric analysis of the specimens, and in the case of Gyrodactylids monogeneans verified by molecular tools.*
**Data source location**	*Mexico: The La Antigua River basin at Veracruz state. Sampled localities (coordinates in decimal degrees) are:* *1. Río Pixquiac, latitude 19.4771266101752, longitude −96.95057920854761; altitude 1245* *m* *2. Xico, 19.41593761847794, −97.00644248792315; 1438* *3. Agua Bendita, 19.407569976733313, −97.0111046464469; 1278* *4. Teocelo, 19.374513314033795, −96.9787067891338; 1115* *5. Baxtla, 19.362335453540677, −96.98019891699604;1105* *6. Jalcomulco, 19.385334362945315, −96.85031307355125; 617* *7. Apazapan, 19.33399614185296, −96.72907308833342; 328* *8. El Carrizal, 19.32069847399925, −96.63299030639037; 211* *9. Río de los Pescados, 19.313584606155054, −96.70170028538092; 282* *10. Puente Nacional, 19.324436302816224, −96.48194616590752; 78* *11. Antigua Presa, 19.342617121064546, −96.42336882872104; 42*
**Data accessibility**	With the article
**Related research article**	Salgado-Maldonado, G. Caspeta-Mandujano, J. M. Mendoza-Franco, E. F. Rubio-Godoy, M. García-Vásquez, A. Mercado-Silva, N. Guzmán-Valdivieso, I. W. Matamoros. Competition from sea to mountain: interactions and aggregation in low diversity monogenean and endohelminth communities in twospot livebearer *Pseudoxiphophorus bimaculatus* (Teleostei: Poeciliidae) populations in a neotropical river. Ecology and Evolution. In Press.

**Value of the Data**

• There are few available raw data regarding helminth parasites of freshwater fishes. The present data can be useful to compare population or community characteristics of helminth parasites, i.e. presence, abundance, spatial distribution, temporal variation, richness of communities.
• These data could be important for parasitologists, helminthologists, ecologists, biogeographers and general zoologists; as well as aquaculturists, veterinarians, conservationists, regulatory agencies and stakeholders who seek to protect the public and their goods or values by limiting the adverse environmental impacts of development.
• These data might be used to examine spatial distribution of helminth parasite taxa. These data migth be reused to examine the spatial variation in community structure of helminth parasites of freshwater fish. These data could support to explore characteristics of the structure assemblages as nestedness or patterns of decay of similarity with distance. And might also assist to compare patterns of structure of assemblage vs. appropriate null models.
• This kind of data could be used to provide an assessment of human environmental impacts, or for public awareness of conservation objectives. Host-parasite system knowledge can be used to indicate changes in parasite biodiversity status [5].

## Data description

The next 18 helminth taxa were recorded from the parasitological examinantion of 220 fish *Pseudoxiphophorus bimaculatus* from 11 localtions along the La Antigua River basin Veracruz, during July 2016. Each helminth taxa is referred with the microhabitat from where it was collected, i.e., the tissue or organ of the fish it parasites. Helminths are referred in a phylogenetical order: Platyhelminthes are first listed then Nematodes. Inside the Platyhelminths, Monogeneans (Class) precede to the Digeneans or Trematodes (Class), and all are referred by Families ordered alphabetically. Adult helminths are first listed and then larvae (metacercariae are the larvae of trematodes). MONOGENEA: Family Dactylogyridae Bychowsky, 1933; *Urocleidoides vaginoclaustroides* Mendoza-Franco, Caspeta-Mandujano, Salgado-Maldonado and Matamoros, 2015 (parasitizing the gills); Family Gyrodactylidae van Beneden and Hesse, 1863**;** *Gyrodactylus takoke* García-Vásquez, Razo-Mendivil and Rubio-Godoy, 2015; *G. xalapensis* Rubio-Godoy, Paladini, García-Vásquez and Shinn, 2010; *Gyrodactylus* sp. (all Gyrodactylids from the fins). TREMATODA, Family Gorgoderidae Looss, 1901 *Phyllodistomum inecoli* Razo-Mendivil, Pérez Ponce de León and Rubio-Godoy, 2013 (parasites of the urinary bladder); Family Allocreadiidae Looss, 1902 *Paracreptotrematoides heterandriae* (Salgado-Maldonado, Caspeta-Mandujano and Vazquez, 2012) (parasites of the intestine); Metacercariae Family Echinostomatidae Looss, 1899**;** *Echinochasmus leopoldinae* Scholz, Ditrich and Vargas-Vázquez, 1996 (from the intestinal mucosa); Family Heterophyidae Odhner, 1914**;** *Centrocestus formosanus* (Nishigori, 1924) (from the gills); *Ascocotyle* (*Leighia*) *megalocephala* Price, 1932 (from the intestinal mucosa); *A*. (*Phagicola*) *macrostoma* (Robinson, 1956) (from the gills); Family Clinostomidae Lühe, 1901 *Clinostomum* cf. *marginatum* Rudolphi, 1819 (from the mesenteries); Family Diplostomidae Poirier, 1886; *Uvulifer ambloplitis* (Hughes, 1927) (from the skin); *Posthodiplostomum* cf. *minimum* (MacCallum, 1921) (from the mesenteries); NEMATODA Family Capillariidae Railliet, 1915**;** *Freitascapillaria moraveci* Caspeta-Mandujano, Salgado-Maldonado and Vázquez, 2009 (from the gall bladder); Family Cystidicolidae Skrjabin, 1946**;** *Spinitectus mexicanus* Caspeta-Mandujano, Moravec and Salgado-Maldonado, 2000 (from the intestine); Nematode larvae, Family Dioctophymatidae Railliet, 1915; *Eustrongylides* sp. (from the mesenteries); Family Anisakidae Railliet and Henry, 1912 *Contracaecum* sp. (from the mesenteries); Family Rhabdochonidae Travassos, Artigas and Pereira, 1928 *Rhabdochona* sp. (from the intestine).

Table 1. Abbreviations and unities used in the data matrix.

**Table 1:** Abbreviations and unities used in the table of data (Table 3).

Abbreviation	Meaning, unities
Lat	Locality_latitude_decimal_degrees
Lon	Locality_longitude_ decimal_degrees
Alt	Locality_altitude_ m_ meters
*H#*	*Host_number*
Tl	Host_Total_length_mm
Sl	Host_Standard_length_mm
Md	Host_Maximum_deep_mm
We	Host_Weight_gr
Sex	Sex_of_host
*Uva*	*Urocleidoides_vaginoclaustrumoides*
*Gta*	*Gyrodactylus_takoke*
*Gxa*	*Gyrodactylus_xalapensis*
*Gyr*	*Gyrodactylus_*sp.
*Pin*	*Phyllodistomum_inecoli*
*Phe*	*Paracreptotrematoides_heterandriae*
*Ele*	*Echinochasmus_leopoldinae*
*Cfo*	*Centrocestus_formosanus*
*Ame*	*Ascocotyle* (*Leighia*) *megalocephala*
*Ama*	*Ascocotyle_*(Phagicola)_*macrostoma*
*Cma*	*Clinostomum_*cf.*_marginatum*
*Uam*	*Uvulifer_amblopitis*
*Pmi*	*Postodiplostomum_minimum*
*Fmo*	*Freitascapillaria_moraveci*
*Sme*	*Spinitectus_mexicanus*
*Eus*	*Eustrongylides*_sp.
*Con*	*Contracaecum_*sp.
*Rha*	*Rhabdochona_*sp.

Table 2. Data set. The data set is contained in a single tex-table including one matrix containing each single host *P. bimaculatus* examined from 11 localities (lines). Measures for each host *P. bimaculatus* include total length, standard length, maximum deep and sex, documented for everyone fish examined, plus data of the number of individual helminth of each taxa collected by each examined fish are placed in the columns. We examined 19 *P bimaculatus* from one locality, 21 from another locality, and 20 from each of the other nine locations sampled in June 2016. A total of 220 individual fish were examined, and in this paper we provide the data for 18 helminth parasite taxa recorded from them. The material in this Data paper comprised the raw data on the abundance, i.e. the number of helminth individuals of each of 18 taxa found in each one individual of *P. bimaculatus* from each of 11 localities.

**Table 2:** Data of helminths of *Pseudoxiphophorus bimaculatus* from 11 localities from La Antigua river, Veracruz, Mexico, recorded in July 2016.

LOCALITY	Lat	Long	Alt	*Host#*	Tl	Sl	Md	We	Sex	*Uva*	*Gta*	*Gxa*	*Gyr*	*Pin*	*Phe*	*Ele*	*Cfo*	*Ame*	*Ama*	*Cma*	*Uam*	*Pmi*	*Fmo*	*Sme*	*Eust*	*Con*	*Rha*
PIXQUIAC	19.477	−96.95	1245	C1	55	48	16	1.6	M	1	NA	NA	2	NA	NA	NA	NA	NA	NA	NA	NA	NA	NA	NA	NA	NA	NA
PIXQUIAC	19.477	−96.95	1245	C2	##	90	30	1.5	F	NA	NA	NA	2	NA	NA	NA	NA	NA	NA	NA	NA	NA	NA	NA	NA	NA	NA
PIXQUIAC	19.477	−96.95	1245	C3	33	25	8	1	F	NA	NA	2	3	NA	NA	NA	NA	NA	NA	NA	NA	NA	NA	NA	NA	NA	NA
PIXQUIAC	19.477	−96.95	1245	C4	35	27	5	0.4	F	NA	NA	NA	2	NA	NA	NA	NA	NA	NA	NA	NA	NA	NA	NA	NA	NA	NA
PIXQUIAC	19.477	−96.95	1245	C5	52	42	12	1.5	M	3	NA	NA	2	NA	NA	NA	NA	NA	NA	NA	NA	NA	NA	NA	NA	NA	NA
PIXQUIAC	19.477	−96.95	1245	C6	62	52	12	2.7	F	2	NA	NA	NA	NA	NA	NA	NA	NA	NA	NA	NA	NA	NA	NA	NA	NA	NA
PIXQUIAC	19.477	−96.95	1245	C7	38	30	7	0.6	F	NA	NA	NA	NA	NA	NA	NA	NA	NA	NA	NA	NA	NA	NA	NA	NA	NA	NA
PIXQUIAC	19.477	−96.95	1245	C8	73	60	18	5.1	F	5	NA	NA	2	NA	NA	NA	NA	NA	NA	NA	NA	NA	NA	NA	NA	NA	NA
PIXQUIAC	19.477	−96.95	1245	C9	60	50	20	3.1	F	NA	1	NA	NA	NA	1	NA	NA	NA	NA	NA	NA	NA	NA	NA	NA	NA	NA
PIXQUIAC	19.477	−96.95	1245	C10	70	58	15	3.6	F	NA	NA	1	NA	NA	NA	NA	NA	NA	NA	NA	NA	NA	NA	NA	NA	NA	NA
PIXQUIAC	19.477	−96.95	1245	C11	37	30	7	0.5	F	1	NA	NA	NA	NA	NA	NA	NA	NA	NA	NA	NA	NA	NA	NA	NA	NA	NA
PIXQUIAC	19.477	−96.95	1245	C12	80	68	20	7	F	NA	NA	NA	NA	NA	NA	NA	NA	NA	NA	NA	NA	NA	NA	NA	NA	NA	NA
PIXQUIAC	19.477	−96.95	1245	C13	50	40	11	1.1	M	4	NA	NA	1	NA	NA	NA	NA	NA	NA	NA	NA	NA	NA	NA	NA	NA	NA
PIXQUIAC	19.477	−96.95	1245	C14	40	34	8	0.7	M	NA	NA	1	2	NA	NA	NA	NA	NA	NA	NA	NA	NA	NA	NA	NA	NA	NA
PIXQUIAC	19.477	−96.95	1245	C15	39	30	8	0.7	F	NA	NA	NA	NA	NA	NA	NA	NA	NA	NA	NA	NA	NA	NA	NA	NA	NA	NA
PIXQUIAC	19.477	−96.95	1245	C16	36	28	8	0.4	F	NA	NA	NA	2	NA	NA	NA	NA	NA	NA	NA	NA	NA	NA	NA	NA	NA	NA
PIXQUIAC	19.477	−96.95	1245	C17	88	72	18	9.1	F	3	NA	NA	NA	NA	NA	NA	NA	NA	NA	NA	NA	NA	NA	NA	NA	NA	NA
PIXQUIAC	19.477	−96.95	1245	C18	34	28	8	1.2	F	NA	NA	1	NA	NA	NA	NA	NA	NA	NA	NA	NA	NA	NA	NA	NA	NA	NA
PIXQUIAC	19.477	−96.95	1245	C19	40	33	7	1.2	M	NA	NA	NA	NA	NA	NA	NA	NA	NA	NA	NA	NA	NA	NA	NA	NA	NA	NA
PIXQUIAC	19.477	−96.95	1245	C20	39	33	7	0.5	F	NA	NA	2	5	NA	NA	NA	NA	NA	NA	NA	NA	NA	NA	NA	NA	NA	NA
XICO	19.415	−97.01	1438	X1	60	50	15	3	F	NA	NA	NA	NA	6	NA	NA	NA	NA	NA	NA	NA	NA	2	NA	NA	NA	NA
XICO	19.415	−97.01	1438	X2	78	65	15	5.1	F	NA	NA	NA	NA	NA	NA	NA	NA	NA	NA	NA	NA	NA	NA	NA	NA	NA	NA
XICO	19.415	−97.01	1438	X3	50	40	9	1.3	F	NA	1	NA	NA	NA	NA	NA	NA	NA	NA	NA	NA	NA	NA	NA	NA	NA	NA
XICO	19.415	−97.01	1438	X4	74	65	28	6.6	F	NA	NA	NA	NA	NA	NA	NA	NA	NA	NA	NA	NA	NA	NA	NA	NA	NA	NA
XICO	19.415	−97.01	1438	X5	42	34	6	0.8	F	NA	1	NA	2	NA	NA	NA	NA	NA	NA	NA	NA	NA	NA	NA	NA	NA	NA
XICO	19.415	−97.01	1438	X6	68	55	15	2.9	F	NA	NA	NA	NA	NA	NA	NA	NA	NA	NA	NA	NA	NA	NA	NA	NA	NA	NA
XICO	19.415	−97.01	1438	X7	67	56	14	3.7	F	NA	1	NA	1	7	NA	NA	NA	NA	NA	NA	NA	NA	NA	NA	NA	NA	NA
XICO	19.415	−97.01	1438	X8	53	44	10	1.5	NA	NA	NA	NA	NA	NA	NA	NA	NA	NA	NA	NA	NA	NA	NA	NA	NA	NA	NA
XICO	19.415	−97.01	1438	X9	50	40	10	1.4	M	NA	NA	NA	NA	1	NA	NA	NA	NA	NA	NA	NA	NA	NA	NA	NA	NA	NA
XICO	19.415	−97.01	1438	X10	42	33	8	0.7	F	NA	1	NA	NA	NA	NA	NA	NA	NA	NA	NA	NA	NA	NA	NA	NA	NA	NA
XICO	19.415	−97.01	1438	X11	50	40	9	1	M	NA	NA	NA	NA	NA	NA	NA	NA	NA	NA	NA	NA	NA	NA	NA	NA	NA	NA
XICO	19.415	−97.01	1438	X12	45	36	8	0.9	F	NA	NA	NA	NA	NA	NA	NA	NA	NA	NA	NA	NA	NA	NA	NA	NA	NA	NA
XICO	19.415	−97.01	1438	X13	48	39	12	1	M	NA	NA	NA	NA	2	NA	NA	NA	NA	NA	NA	NA	NA	NA	NA	NA	NA	NA
XICO	19.415	−97.01	1438	X14	42	33	9	1	F	NA	NA	NA	NA	NA	NA	NA	NA	NA	NA	NA	NA	NA	NA	NA	NA	NA	NA
XICO	19.415	−97.01	1438	X15	32	26	6	0.4	F	NA	1	NA	NA	NA	NA	NA	NA	NA	NA	NA	NA	NA	NA	NA	NA	NA	NA
XICO	19.415	−97.01	1438	X16	52	40	10	1.2	M	NA	NA	NA	NA	NA	NA	NA	NA	NA	NA	NA	NA	NA	NA	NA	NA	NA	NA
XICO	19.415	−97.01	1438	X17	40	32	6	0.7	F	NA	NA	NA	NA	1	NA	NA	NA	NA	NA	NA	NA	NA	NA	NA	NA	NA	NA
XICO	19.415	−97.01	1438	X18	42	37	7	0.6	M	NA	NA	NA	2	NA	NA	NA	NA	NA	NA	NA	NA	NA	NA	NA	NA	NA	NA
XICO	19.415	−97.01	1438	X19	30	25	7	0.4	F	NA	2	NA	3	NA	NA	NA	NA	NA	NA	NA	NA	NA	NA	NA	NA	NA	NA
XICO	19.415	−97.01	1438	X20	35	28	8	0.4	M	NA	NA	NA	NA	NA	NA	NA	NA	NA	NA	NA	NA	NA	NA	NA	NA	NA	NA
AGUA_BENDITA	19.407	−97.01	1278	A1	80	68	20	6.5	F	3	NA	NA	NA	3	NA	NA	NA	NA	NA	NA	NA	NA	7	1	NA	NA	NA
AGUA_BENDITA	19.407	−97.01	1278	A2	NA	33	10	1.2	M	10	NA	1	1	NA	NA	NA	NA	NA	NA	NA	NA	NA	1	NA	NA	NA	NA
AGUA_BENDITA	19.407	−97.01	1278	A3	56	46	10	1.7	M	NA	NA	NA	NA	NA	NA	NA	NA	NA	NA	NA	NA	NA	1	NA	1	NA	NA
AGUA_BENDITA	19.407	−97.01	1278	A4	70	58	15	2.5	F	8	NA	NA	NA	NA	NA	NA	NA	NA	NA	NA	NA	NA	NA	1	NA	NA	NA
AGUA_BENDITA	19.407	−97.01	1278	A5	48	38	9	1	M	13	NA	NA	1	NA	NA	NA	NA	NA	NA	NA	NA	NA	2	NA	NA	NA	NA
AGUA_BENDITA	19.407	−97.01	1278	A6	58	48	11	1.9	F	14	NA	NA	NA	1	NA	NA	NA	NA	NA	NA	NA	NA	2	1	NA	NA	NA
AGUA_BENDITA	19.407	−97.01	1278	A7	64	55	12	2.5	F	NA	NA	1	NA	NA	NA	NA	NA	NA	NA	NA	NA	NA	2	NA	NA	NA	NA
AGUA_BENDITA	19.407	−97.01	1278	A8	75	60	20	6.9	F	4	NA	NA	NA	NA	NA	NA	NA	NA	NA	NA	NA	NA	NA	2	NA	NA	NA
AGUA_BENDITA	19.407	−97.01	1278	A9	70	60	19	5.7	F	28	NA	NA	NA	6	NA	NA	NA	NA	NA	NA	NA	NA	2	NA	NA	NA	NA
AGUA_BENDITA	19.407	−97.01	1278	A10	55	45	8	1.5	M	NA	1	NA	NA	NA	NA	NA	NA	NA	NA	NA	NA	NA	NA	NA	NA	NA	NA
AGUA_BENDITA	19.407	−97.01	1278	A11	70	60	16	4.2	F	4	NA	NA	NA	5	NA	NA	NA	NA	NA	NA	NA	NA	4	1	NA	NA	NA
AGUA_BENDITA	19.407	−97.01	1278	A12	57	47	10	1.8	M	NA	NA	NA	NA	NA	NA	NA	NA	NA	NA	NA	NA	NA	1	NA	NA	NA	NA
AGUA_BENDITA	19.407	−97.01	1278	A13	70	57	13	3.3	F	NA	1	NA	NA	NA	NA	NA	NA	NA	NA	NA	NA	NA	3	2	NA	NA	NA
AGUA_BENDITA	19.407	−97.01	1278	A14	55	48	13	1.2	F	2	NA	NA	1	NA	NA	NA	NA	NA	NA	NA	NA	NA	1	NA	NA	NA	NA
AGUA_BENDITA	19.407	−97.01	1278	A15	50	41	10	1.5	F	8	1	NA	1	NA	NA	NA	NA	NA	NA	NA	NA	NA	NA	NA	NA	NA	NA
AGUA_BENDITA	19.407	−97.01	1278	A16	55	45	10	1.5	M	8	NA	NA	NA	10	NA	NA	NA	NA	NA	NA	NA	NA	3	NA	NA	NA	NA
AGUA_BENDITA	19.407	−97.01	1278	A18	51	44	10	1.2	M	10	NA	NA	NA	NA	NA	NA	NA	NA	NA	NA	NA	NA	2	NA	NA	NA	NA
AGUA_BENDITA	19.407	−97.01	1278	A19	65	54	15	3.9	F	6	NA	NA	NA	NA	NA	NA	NA	NA	NA	NA	NA	NA	2	NA	NA	NA	NA
AGUA_BENDITA	19.407	−97.01	1278	A20	55	44	13	2	M	13	NA	NA	NA	NA	NA	NA	NA	NA	NA	NA	NA	NA	3	NA	NA	NA	NA
TEOCELO	19.374	−96.98	1115	T1	66	55	12	2.7	F	8	NA	NA	7	NA	2	NA	NA	NA	NA	NA	NA	NA	NA	1	NA	NA	NA
TEOCELO	19.374	−96.98	1115	T2	75	65	15	5.2	F	21	NA	NA	9	NA	NA	NA	NA	NA	NA	NA	NA	NA	NA	NA	NA	NA	NA
TEOCELO	19.374	−96.98	1115	T3	38	30	8	1.4	F	NA	1	NA	NA	NA	NA	NA	NA	NA	NA	NA	NA	NA	NA	NA	NA	NA	NA
TEOCELO	19.374	−96.98	1115	T4	48	37	10	1	M	3	NA	NA	2	5	NA	NA	NA	NA	NA	NA	NA	NA	NA	NA	NA	NA	NA
TEOCELO	19.374	−96.98	1115	T5	60	50	13	2	F	8	3	7	4	NA	NA	NA	NA	NA	NA	NA	NA	NA	NA	4	NA	NA	NA
TEOCELO	19.374	−96.98	1115	T6	48	40	9	1	F	NA	NA	NA	3	NA	NA	NA	NA	NA	NA	NA	NA	NA	NA	NA	NA	NA	NA
TEOCELO	19.374	−96.98	1115	T7	65	55	15	3.3	NA	25	2	3	11	NA	NA	NA	NA	NA	NA	NA	NA	NA	NA	NA	NA	NA	NA
TEOCELO	19.374	−96.98	1115	T8	81	70	28	6.8	F	26	NA	NA	4	NA	NA	NA	NA	NA	NA	NA	NA	NA	NA	NA	NA	NA	NA
TEOCELO	19.374	−96.98	1115	T9	61	50	14	2.3	M	NA	NA	NA	NA	3	NA	NA	NA	NA	NA	NA	NA	NA	NA	1	NA	NA	NA
TEOCELO	19.374	−96.98	1115	T10	52	43	18	1.5	M	3	NA	2	1	NA	NA	NA	NA	NA	NA	NA	NA	NA	NA	NA	NA	NA	NA
TEOCELO	19.374	−96.98	1115	T11	74	60	15	4.6	NA	17	NA	NA	6	NA	NA	NA	NA	NA	NA	NA	NA	NA	NA	NA	NA	NA	NA
TEOCELO	19.374	−96.98	1115	T12	65	60	15	3.5	F	NA	NA	NA	6	3	1	NA	NA	NA	NA	NA	NA	NA	NA	1	NA	NA	NA
TEOCELO	19.374	−96.98	1115	T13	59	48	25	1.8	F	3	NA	NA	8	NA	NA	NA	NA	NA	NA	NA	NA	NA	NA	NA	NA	NA	NA
TEOCELO	19.374	−96.98	1115	T14	60	50	15	2.4	M	6	NA	NA	5	2	NA	NA	NA	NA	NA	NA	NA	NA	NA	NA	NA	NA	NA
TEOCELO	19.374	−96.98	1115	T15	46	37	9	1.3	F	11	3	2	15	NA	NA	NA	NA	NA	NA	NA	NA	NA	NA	NA	NA	NA	NA
TEOCELO	19.374	−96.98	1115	T16	53	43	16	1.7	M	5	1	4	6	4	NA	NA	NA	NA	NA	NA	NA	NA	NA	NA	NA	NA	NA
TEOCELO	19.374	−96.98	1115	T17	60	50	18	1.7	M	5	NA	NA	5	8	NA	NA	NA	NA	NA	NA	NA	NA	NA	NA	NA	NA	NA
TEOCELO	19.374	−96.98	1115	T18	61	52	12	2.7	F	NA	NA	NA	9	1	NA	NA	NA	NA	NA	NA	NA	NA	NA	NA	NA	NA	NA
TEOCELO	19.374	−96.98	1115	T19	58	53	14	2.4	M	7	2	2	NA	NA	NA	NA	NA	NA	NA	NA	NA	NA	NA	1	NA	NA	NA
TEOCELO	19.374	−96.98	1115	T20	67	56	15	3.2	F	2	NA	1	NA	3	NA	NA	NA	NA	NA	NA	NA	NA	NA	2	NA	NA	NA
BAXTLA	19.362	−96.98	1105	B1	72	61	16	4.5	F	18	4	1	6	10	NA	NA	NA	NA	NA	NA	NA	NA	NA	NA	NA	NA	NA
BAXTLA	19.362	−96.98	1105	B2	92	80	25	12.6	F	84	2	1	4	NA	NA	NA	NA	NA	NA	NA	NA	NA	NA	NA	NA	NA	NA
BAXTLA	19.362	−96.98	1105	B3	59	48	13	2.7	F	8	NA	NA	2	NA	NA	NA	NA	NA	NA	NA	NA	NA	NA	NA	NA	NA	NA
BAXTLA	19.362	−96.98	1105	B4	60	50	14	1.9	M	11	2	NA	5	NA	NA	NA	NA	NA	NA	NA	3	NA	NA	NA	NA	NA	NA
BAXTLA	19.362	−96.98	1105	B5	59	49	12	1.7	M	4	NA	NA	4	NA	NA	NA	NA	NA	NA	NA	NA	NA	NA	NA	NA	NA	NA
BAXTLA	19.362	−96.98	1105	B6	95	83	25	12.3	F	73	NA	NA	3	NA	2	NA	NA	NA	NA	NA	NA	NA	NA	NA	NA	NA	NA
BAXTLA	19.362	−96.98	1105	B7	NA	65	20	5.7	F	5	3	2	4	NA	NA	NA	NA	NA	NA	NA	NA	NA	NA	NA	NA	NA	NA
BAXTLA	19.362	−96.98	1105	B8	66	55	14	5.5	F	12	1	2	4	6	NA	NA	NA	NA	NA	NA	NA	NA	NA	NA	NA	NA	NA
BAXTLA	19.362	−96.98	1105	B9	55	47	10	1.4	M	NA	NA	NA	6	NA	1	NA	NA	NA	NA	NA	NA	NA	NA	NA	NA	NA	NA
BAXTLA	19.362	−96.98	1105	B10	40	30	10	1.2	M	2	NA	NA	5	7	NA	NA	NA	NA	NA	NA	NA	NA	NA	NA	NA	NA	NA
BAXTLA	19.362	−96.98	1105	B11	85	80	17	6.8	M	NA	NA	5	5	NA	NA	NA	NA	NA	NA	NA	NA	NA	NA	NA	NA	NA	NA
BAXTLA	19.362	−96.98	1105	B12	80	70	19	6	F	19	NA	NA	3	6	NA	NA	NA	NA	NA	NA	NA	NA	NA	NA	NA	NA	NA
BAXTLA	19.362	−96.98	1105	B13	55	45	10	1.2	M	8	NA	NA	4	NA	NA	NA	NA	NA	NA	NA	NA	NA	NA	NA	NA	NA	NA
BAXTLA	19.362	−96.98	1105	B14	47	38	10	0.7	M	2	NA	NA	4	NA	NA	NA	NA	NA	NA	NA	NA	NA	NA	NA	NA	NA	NA
BAXTLA	19.362	−96.98	1105	B15	45	40	9	1	M	NA	1	NA	NA	NA	NA	NA	NA	NA	NA	NA	NA	NA	NA	NA	NA	NA	NA
BAXTLA	19.362	−96.98	1105	B16	47	38	10	1	F	3	NA	NA	1	NA	NA	NA	NA	NA	NA	NA	NA	NA	NA	NA	NA	NA	NA
BAXTLA	19.362	−96.98	1105	B17	42	35	9	0.7	M	NA	NA	2	NA	NA	NA	NA	NA	NA	NA	NA	NA	NA	NA	NA	NA	NA	NA
BAXTLA	19.362	−96.98	1105	B18	43	33	8	0.5	M	5	2	3	6	NA	NA	NA	NA	NA	NA	NA	NA	1	NA	NA	NA	NA	NA
BAXTLA	19.362	−96.98	1105	B19	50	40	10	1.3	M	9	NA	NA	2	NA	NA	NA	NA	NA	NA	NA	NA	NA	NA	NA	NA	NA	NA
BAXTLA	19.362	−96.98	1105	B20	46	39	10	1.1	NA	7	NA	NA	3	NA	1	NA	NA	NA	NA	NA	NA	NA	NA	NA	NA	NA	NA
JALCOMULCO	19.385	−96.85	617	J1	77	65	15	3.8	F	7	1	NA	NA	NA	NA	NA	11	1	NA	NA	NA	10	1	NA	NA	NA	NA
JALCOMULCO	19.385	−96.85	617	J2	50	40	10	1.4	F	NA	NA	NA	NA	NA	NA	NA	160	NA	NA	NA	2	3	NA	NA	NA	NA	NA
JALCOMULCO	19.385	−96.85	617	J3	47	39	11	1	M	NA	NA	NA	NA	NA	1	NA	22	NA	NA	2	NA	NA	3	1	NA	NA	NA
JALCOMULCO	19.385	−96.85	617	J4	54	44	15	1.4	M	3	1	NA	NA	8	20	NA	NA	NA	NA	NA	5	1	NA	4	NA	NA	NA
JALCOMULCO	19.385	−96.85	617	J5	56	46	11	2.3	F	5	NA	NA	NA	NA	NA	NA	NA	NA	NA	1	3	NA	7	NA	NA	NA	NA
JALCOMULCO	19.385	−96.85	617	J6	52	43	10	1.4	F	7	NA	NA	NA	NA	NA	2	9	NA	NA	NA	2	1	6	NA	NA	NA	NA
JALCOMULCO	19.385	−96.85	617	J7	52	43	11	1.5	F	2	NA	NA	NA	NA	NA	NA	15	NA	NA	NA	6	3	5	NA	NA	NA	NA
JALCOMULCO	19.385	−96.85	617	J8	64	52	13	2.6	F	NA	NA	NA	NA	NA	32	NA	25	NA	NA	NA	4	NA	NA	NA	NA	NA	NA
JALCOMULCO	19.385	−96.85	617	J9	75	60	20	5.1	F	NA	NA	NA	NA	3	5	3	344	NA	NA	NA	NA	1	7	2	NA	NA	NA
JALCOMULCO	19.385	−96.85	617	J10	62	52	15	3.5	F	3	NA	NA	NA	9	4	30	25	NA	NA	NA	NA	1	NA	NA	NA	NA	NA
JALCOMULCO	19.385	−96.85	617	J11	57	47	12	2.3	F	17	NA	NA	NA	NA	4	NA	2	NA	NA	NA	NA	NA	1	NA	NA	NA	NA
JALCOMULCO	19.385	−96.85	617	J12	51	44	10	1.2	M	NA	NA	NA	NA	NA	1	NA	37	NA	NA	NA	2	18	NA	5	NA	NA	NA
JALCOMULCO	19.385	−96.85	617	J13	70	56	14	3.7	F	NA	NA	NA	1	NA	20	NA	12	NA	NA	NA	5	NA	NA	6	NA	NA	NA
JALCOMULCO	19.385	−96.85	617	J14	50	40	12	1.6	F	NA	2	NA	1	NA	NA	NA	NA	NA	NA	NA	5	NA	2	NA	NA	NA	NA
JALCOMULCO	19.385	−96.85	617	J15	52	41	10	1.5	M	NA	NA	NA	NA	NA	NA	NA	4	NA	NA	NA	1	NA	5	NA	NA	NA	NA
JALCOMULCO	19.385	−96.85	617	J16	50	40	12	1.4	F	1	NA	NA	NA	NA	NA	NA	15	NA	NA	NA	1	NA	5	NA	NA	NA	NA
JALCOMULCO	19.385	−96.85	617	J17	56	47	12	2.1	F	1	NA	NA	NA	NA	NA	2	17	NA	NA	NA	NA	NA	NA	3	NA	NA	NA
JALCOMULCO	19.385	−96.85	617	J18	52	42	16	1.6	M	NA	1	1	1	NA	1	NA	23	NA	NA	NA	NA	NA	6	2	NA	NA	NA
JALCOMULCO	19.385	−96.85	617	J19	45	35	10	1.3	F	1	NA	NA	NA	NA	NA	1	23	NA	NA	NA	4	NA	9	NA	NA	NA	NA
JALCOMULCO	19.385	−96.85	617	J20	50	42	13	1.7	M	5	1	NA	NA	NA	NA	NA	6	NA	NA	NA	NA	1	NA	NA	NA	NA	NA
APAZAPAN	19.333	−96.73	328	Z1	53	43	10	3.5	F	NA	NA	NA	NA	NA	2	NA	NA	NA	1	NA	NA	NA	3	NA	NA	NA	NA
APAZAPAN	19.333	−96.73	328	Z2	55	45	10	1.7	M	NA	NA	NA	NA	NA	NA	NA	NA	NA	NA	NA	NA	NA	1	NA	NA	NA	NA
APAZAPAN	19.333	−96.73	328	Z3	35	28	7	0.5	M	NA	NA	NA	NA	NA	NA	NA	1	NA	NA	NA	NA	NA	NA	NA	NA	NA	NA
APAZAPAN	19.333	−96.73	328	Z4	54	43	8	1.5	M	NA	NA	NA	NA	1	NA	NA	NA	NA	NA	NA	NA	NA	1	NA	NA	NA	NA
APAZAPAN	19.333	−96.73	328	Z5	42	33	10	0.7	M	NA	NA	NA	NA	NA	NA	NA	2	NA	NA	NA	NA	NA	2	NA	NA	NA	1
APAZAPAN	19.333	−96.73	328	Z6	60	55	13	2.6	F	4	NA	NA	NA	NA	NA	NA	NA	NA	NA	NA	NA	NA	2	NA	NA	NA	NA
APAZAPAN	19.333	−96.73	328	Z7	42	33	10	0.7	M	2	NA	NA	NA	NA	NA	NA	NA	NA	NA	NA	NA	NA	1	NA	NA	NA	NA
APAZAPAN	19.333	−96.73	328	Z8	50	40	10	1	M	1	NA	NA	NA	NA	4	NA	NA	NA	NA	NA	NA	NA	NA	NA	NA	NA	NA
APAZAPAN	19.333	−96.73	328	Z9	45	35	9	0.7	NA	2	NA	NA	NA	NA	1	NA	NA	4	NA	NA	2	NA	2	NA	NA	NA	NA
APAZAPAN	19.333	−96.73	328	Z10	45	36	10	0.9	M	3	NA	NA	NA	NA	1	NA	NA	1	NA	2	NA	NA	NA	NA	NA	NA	NA
APAZAPAN	19.333	−96.73	328	Z11	52	41	10	1.3	F	2	NA	NA	NA	NA	NA	NA	2	NA	NA	NA	NA	NA	1	NA	NA	NA	NA
APAZAPAN	19.333	−96.73	328	Z12	45	36	10	0.9	M	2	NA	NA	NA	NA	NA	NA	1	NA	NA	NA	NA	NA	1	NA	NA	NA	NA
APAZAPAN	19.333	−96.73	328	Z13	32	25	5	0.3	M	NA	NA	NA	NA	NA	NA	NA	NA	NA	NA	NA	NA	NA	NA	NA	NA	NA	NA
APAZAPAN	19.333	−96.73	328	Z14	37	30	9	0.6	F	2	NA	NA	NA	NA	1	NA	NA	NA	NA	NA	NA	NA	NA	NA	NA	NA	NA
APAZAPAN	19.333	−96.73	328	Z15	42	33	8	0.5	M	2	NA	NA	NA	2	NA	NA	NA	NA	NA	NA	NA	NA	NA	NA	NA	NA	NA
APAZAPAN	19.333	−96.73	328	Z16	46	36	10	1	M	6	NA	NA	NA	NA	NA	NA	NA	NA	NA	NA	NA	NA	2	NA	NA	NA	NA
APAZAPAN	19.333	−96.73	328	Z17	42	33	9	0.6	F	8	NA	NA	NA	NA	1	NA	NA	NA	NA	NA	NA	NA	1	NA	NA	NA	NA
APAZAPAN	19.333	−96.73	328	Z18	35	27	8	0.4	F	14	NA	NA	NA	NA	NA	NA	NA	NA	NA	NA	NA	NA	NA	NA	NA	NA	NA
APAZAPAN	19.333	−96.73	328	Z19	33	28	5	0.3	F	NA	NA	NA	NA	NA	NA	NA	NA	NA	NA	NA	NA	NA	NA	NA	NA	NA	NA
APAZAPAN	19.333	−96.73	328	Z20	47	38	9	1	F	2	NA	NA	NA	NA	3	NA	NA	NA	NA	NA	NA	NA	NA	NA	NA	NA	NA
RÍO_DE_LOS_PESCADOS	19.313	−96.7	282	P1	60	50	14	2.6	F	1	NA	NA	NA	NA	NA	NA	NA	NA	NA	NA	NA	NA	NA	NA	NA	NA	NA
RÍO_DE_LOS_PESCADOS	19.313	−96.7	282	P2	60	51	14	2.7	F	NA	NA	NA	1	NA	NA	NA	NA	NA	NA	NA	1	NA	NA	NA	NA	NA	NA
RÍO_DE_LOS_PESCADOS	19.313	−96.7	282	P3	62	48	12	2	F	NA	NA	NA	NA	NA	NA	NA	NA	NA	NA	NA	NA	NA	NA	NA	NA	NA	NA
RÍO_DE_LOS_PESCADOS	19.313	−96.7	282	P4	48	38	11	1.2	M	NA	NA	NA	2	NA	NA	NA	NA	NA	NA	NA	NA	NA	NA	NA	NA	NA	NA
RÍO_DE_LOS_PESCADOS	19.313	−96.7	282	P5	65	53	11	2.8	F	NA	NA	NA	1	NA	NA	NA	NA	NA	NA	NA	NA	NA	NA	NA	NA	NA	NA
RÍO_DE_LOS_PESCADOS	19.313	−96.7	282	P6	55	45	12	1.9	F	2	NA	NA	NA	NA	NA	NA	NA	NA	NA	NA	NA	NA	NA	NA	NA	NA	NA
RÍO_DE_LOS_PESCADOS	19.313	−96.7	282	P7	41	33	7	0.6	M	2	NA	NA	NA	NA	NA	NA	NA	NA	NA	NA	NA	NA	NA	NA	NA	NA	NA
RÍO_DE_LOS_PESCADOS	19.313	−96.7	282	P8	NA	NA	NA	2.1	M	1	1	NA	2	NA	NA	NA	NA	NA	NA	NA	NA	NA	NA	NA	NA	NA	NA
RÍO_DE_LOS_PESCADOS	19.313	−96.7	282	P9	58	45	11	2.1	M	NA	NA	NA	2	NA	NA	NA	NA	NA	NA	NA	NA	NA	NA	NA	NA	NA	NA
RÍO_DE_LOS_PESCADOS	19.313	−96.7	282	P10	63	59	13	3.2	F	NA	NA	1	NA	NA	NA	NA	NA	NA	NA	NA	NA	NA	NA	NA	NA	NA	NA
RÍO_DE_LOS_PESCADOS	19.313	−96.7	282	P11	50	40	10	1.2	M	NA	NA	NA	NA	NA	NA	NA	NA	NA	NA	NA	NA	NA	NA	NA	NA	NA	NA
RÍO_DE_LOS_PESCADOS	19.313	−96.7	282	P12	52	42	10	1.7	M	NA	NA	NA	NA	NA	NA	NA	NA	NA	NA	NA	NA	NA	NA	NA	NA	NA	NA
RÍO_DE_LOS_PESCADOS	19.313	−96.7	282	P13	52	43	10	1.4	F	NA	NA	NA	NA	NA	NA	NA	NA	NA	NA	NA	NA	NA	NA	NA	NA	NA	NA
RÍO_DE_LOS_PESCADOS	19.313	−96.7	282	P14	50	40	12	1.3	M	1	1	NA	NA	NA	NA	NA	NA	NA	NA	NA	NA	NA	NA	NA	NA	NA	NA
RÍO_DE_LOS_PESCADOS	19.313	−96.7	282	P15	45	38	8	0.8	F	NA	NA	NA	NA	NA	NA	NA	NA	NA	NA	NA	NA	NA	NA	NA	NA	NA	NA
RÍO_DE_LOS_PESCADOS	19.313	−96.7	282	P16	40	32	8	0.7	M	NA	NA	NA	NA	NA	NA	NA	NA	NA	NA	NA	NA	NA	NA	NA	NA	NA	NA
RÍO_DE_LOS_PESCADOS	19.313	−96.7	282	P17	51	42	10	1.3	F	NA	1	NA	3	NA	NA	NA	NA	NA	NA	NA	NA	NA	NA	NA	NA	NA	NA
RÍO_DE_LOS_PESCADOS	19.313	−96.7	282	P18	87	70	20	7	F	NA	NA	NA	NA	NA	NA	NA	NA	NA	NA	NA	NA	NA	NA	NA	NA	NA	NA
RÍO_DE_LOS_PESCADOS	19.313	−96.7	282	P19	48	39	9	0.9	F	NA	2	1	1	NA	NA	NA	NA	NA	NA	NA	NA	NA	NA	NA	NA	NA	NA
RÍO_DE_LOS_PESCADOS	19.313	−96.7	282	P20	65	55	14	2.6	F	NA	NA	NA	NA	NA	NA	NA	NA	NA	NA	NA	NA	NA	NA	NA	NA	NA	NA
EL_CARRIZAL	19.32	−96.63	211	RR1	NA	NA	NA	1.6	M	NA	NA	NA	NA	NA	NA	NA	NA	NA	NA	NA	NA	NA	NA	NA	NA	NA	NA
EL_CARRIZAL	19.32	−96.63	211	RR2	54	44	13	2.5	M	2	NA	NA	NA	NA	NA	NA	NA	NA	NA	NA	NA	NA	NA	NA	NA	NA	NA
EL_CARRIZAL	19.32	−96.63	211	RR3	44	35	10	0.8	M	NA	NA	NA	NA	NA	NA	NA	NA	NA	NA	NA	NA	NA	NA	NA	NA	NA	NA
EL_CARRIZAL	19.32	−96.63	211	RR4	52	42	10	1.5	F	NA	NA	NA	NA	NA	NA	NA	NA	NA	NA	NA	NA	NA	NA	NA	NA	NA	NA
EL_CARRIZAL	19.32	−96.63	211	RR5	48	39	9	1	NA	3	NA	NA	NA	NA	NA	NA	NA	NA	NA	NA	3	3	NA	NA	NA	NA	NA
EL_CARRIZAL	19.32	−96.63	211	RR6	60	48	12	2.8	F	NA	NA	NA	NA	NA	NA	NA	NA	NA	NA	NA	NA	NA	NA	NA	NA	NA	NA
EL_CARRIZAL	19.32	−96.63	211	RR7	49	39	13	1.6	M	NA	1	NA	NA	NA	NA	NA	NA	NA	NA	NA	NA	NA	NA	NA	NA	NA	NA
EL_CARRIZAL	19.32	−96.63	211	RR8	55	47	10	1.4	M	NA	NA	1	NA	NA	NA	NA	NA	NA	NA	NA	NA	NA	NA	NA	NA	NA	NA
EL_CARRIZAL	19.32	−96.63	211	RR9	42	34	9	0.8	M	NA	NA	NA	NA	NA	NA	NA	NA	NA	NA	NA	NA	NA	NA	NA	NA	NA	NA
EL_CARRIZAL	19.32	−96.63	211	RR10	70	61	14	3.5	F	NA	NA	2	1	NA	NA	NA	NA	NA	NA	NA	NA	NA	NA	NA	NA	NA	NA
EL_CARRIZAL	19.32	−96.63	211	RR11	67	56	14	2.9	NA	NA	NA	NA	NA	NA	NA	NA	1	NA	NA	NA	NA	NA	NA	NA	NA	NA	NA
EL_CARRIZAL	19.32	−96.63	211	RR12	51	42	10	1.2	M	NA	NA	NA	NA	NA	NA	NA	37	NA	NA	NA	NA	NA	NA	NA	NA	NA	NA
EL_CARRIZAL	19.32	−96.63	211	RR13	38	30	8	0.7	M	NA	NA	1	NA	NA	NA	NA	NA	NA	NA	NA	NA	NA	NA	NA	NA	NA	NA
EL_CARRIZAL	19.32	−96.63	211	RR14	42	35	9	0.6	M	NA	NA	NA	NA	NA	NA	NA	NA	NA	NA	NA	NA	NA	NA	NA	NA	NA	NA
EL_CARRIZAL	19.32	−96.63	211	RR15	40	33	9	0.8	F	NA	NA	NA	NA	NA	NA	NA	NA	NA	NA	NA	NA	NA	NA	NA	NA	NA	NA
EL_CARRIZAL	19.32	−96.63	211	RR16	45	35	10	0.9	M	NA	NA	NA	NA	NA	NA	NA	NA	NA	NA	NA	NA	NA	NA	NA	NA	NA	NA
EL_CARRIZAL	19.32	−96.63	211	RR17	40	32	6	0.6	F	NA	NA	NA	NA	NA	NA	NA	NA	NA	NA	NA	NA	NA	NA	NA	NA	NA	NA
EL_CARRIZAL	19.32	−96.63	211	RR18	40	31	9	0.5	F	NA	NA	1	NA	NA	NA	NA	NA	NA	NA	NA	NA	NA	NA	NA	NA	NA	NA
EL_CARRIZAL	19.32	−96.63	211	RR19	43	35	8	0.9	F	NA	NA	1	NA	NA	NA	NA	NA	NA	NA	NA	NA	NA	NA	NA	NA	NA	NA
EL_CARRIZAL	19.32	−96.63	211	RR20	56	45	13	2	M	NA	NA	NA	1	NA	NA	NA	NA	NA	NA	NA	NA	NA	NA	NA	NA	NA	NA
PUENTE_NACIONAL	19.324	−96.48	78	PN1	78	73	18	6.7	F	NA	NA	NA	NA	NA	NA	NA	NA	NA	NA	NA	NA	NA	NA	NA	NA	NA	NA
PUENTE_NACIONAL	19.324	−96.48	78	PN2	58	49	10	1.9	M	NA	NA	NA	NA	NA	NA	NA	NA	NA	NA	NA	NA	NA	NA	NA	NA	NA	NA
PUENTE_NACIONAL	19.324	−96.48	78	PN3	55	45	12	2.5	F	NA	NA	NA	1	NA	NA	NA	NA	NA	NA	NA	NA	NA	NA	NA	NA	NA	NA
PUENTE_NACIONAL	19.324	−96.48	78	PN4	46	38	10	1	F	NA	NA	NA	NA	NA	NA	NA	NA	NA	NA	NA	NA	NA	NA	NA	NA	NA	NA
PUENTE_NACIONAL	19.324	−96.48	78	PN5	48	37	9	1.1	F	NA	NA	NA	NA	NA	NA	NA	NA	NA	NA	NA	NA	NA	NA	NA	NA	NA	NA
PUENTE_NACIONAL	19.324	−96.48	78	PN6	65	56	12	3	F	1	NA	NA	NA	NA	NA	NA	1	NA	NA	NA	NA	NA	NA	NA	NA	NA	NA
PUENTE_NACIONAL	19.324	−96.48	78	PN7	65	54	13	3.4	F	NA	NA	NA	1	NA	NA	NA	NA	NA	NA	NA	NA	NA	NA	NA	NA	NA	NA
PUENTE_NACIONAL	19.324	−96.48	78	PN8	45	38	9	0.7	M	NA	NA	1	NA	NA	NA	NA	NA	NA	NA	NA	NA	NA	NA	NA	NA	NA	NA
PUENTE_NACIONAL	19.324	−96.48	78	PN9	51	42	12	1.7	M	NA	NA	NA	NA	NA	NA	NA	NA	NA	NA	NA	NA	NA	NA	NA	NA	NA	NA
PUENTE_NACIONAL	19.324	−96.48	78	PN10	42	35	10	1	F	2	NA	NA	NA	NA	NA	NA	NA	NA	NA	NA	NA	NA	NA	NA	NA	NA	NA
PUENTE_NACIONAL	19.324	−96.48	78	PN11	55	45	12	1.8	M	1	NA	NA	NA	NA	NA	NA	NA	NA	NA	NA	NA	NA	NA	NA	NA	NA	NA
PUENTE_NACIONAL	19.324	−96.48	78	PN12	56	46	13	1.8	F	1	NA	NA	1	NA	NA	NA	NA	NA	NA	NA	NA	NA	NA	NA	NA	NA	NA
PUENTE_NACIONAL	19.324	−96.48	78	PN13	41	33	10	0.7	M	1	NA	NA	NA	NA	NA	NA	NA	NA	NA	NA	NA	NA	NA	NA	NA	NA	NA
PUENTE_NACIONAL	19.324	−96.48	78	PN14	58	46	15	1.9	M	NA	NA	NA	NA	NA	NA	NA	NA	NA	NA	NA	NA	NA	NA	NA	NA	NA	NA
PUENTE_NACIONAL	19.324	−96.48	78	PN14´	49	40	10	1.3	M	NA	NA	NA	NA	NA	NA	NA	NA	NA	NA	NA	NA	NA	NA	NA	NA	NA	NA
PUENTE_NACIONAL	19.324	−96.48	78	PN15	50	41	11	1.7	F	NA	NA	NA	NA	NA	NA	NA	NA	NA	NA	NA	NA	NA	NA	NA	NA	NA	NA
PUENTE_NACIONAL	19.324	−96.48	78	PN16	47	38	9	1.3	F	NA	2	2	NA	NA	NA	NA	NA	NA	NA	NA	NA	NA	NA	NA	NA	NA	NA
PUENTE_NACIONAL	19.324	−96.48	78	PN17	34	28	5	0.3	F	NA	1	NA	NA	NA	NA	NA	NA	NA	NA	NA	NA	NA	NA	NA	NA	NA	NA
PUENTE_NACIONAL	19.324	−96.48	78	PN18	55	42	12	1.2	M	NA	NA	NA	NA	NA	NA	NA	NA	NA	NA	NA	NA	NA	NA	NA	NA	NA	NA
PUENTE_NACIONAL	19.324	−96.48	78	PN19	NA	NA	NA	0.3	F	NA	NA	NA	NA	NA	NA	NA	NA	NA	NA	NA	NA	NA	NA	NA	NA	NA	NA
PUENTE_NACIONAL	19.324	−96.48	78	PN20	40	32	8	1	F	NA	NA	1	NA	NA	NA	NA	NA	NA	NA	NA	NA	NA	NA	NA	NA	NA	1
ANTIGUA_PRESA	19.342	−96.42	42	AP1	33	27	5	0.4	F	NA	NA	NA	NA	NA	NA	NA	NA	NA	NA	NA	NA	NA	NA	NA	NA	NA	NA
ANTIGUA_PRESA	19.342	−96.42	42	AP2	62	51	12	2.8	F	NA	NA	NA	NA	NA	NA	NA	NA	NA	NA	NA	NA	NA	NA	NA	NA	NA	NA
ANTIGUA_PRESA	19.342	−96.42	42	AP3	57	48	9	1.8	F	6	NA	NA	NA	NA	NA	NA	4	NA	NA	NA	NA	1	NA	NA	NA	3	NA
ANTIGUA_PRESA	19.342	−96.42	42	AP4	49	40	11	1	M	NA	NA	NA	NA	NA	NA	NA	NA	NA	NA	NA	NA	NA	NA	NA	NA	1	NA
ANTIGUA_PRESA	19.342	−96.42	42	AP5	36	23	4	0.3	F	2	NA	NA	NA	NA	NA	NA	NA	NA	NA	NA	NA	NA	NA	NA	NA	1	NA
ANTIGUA_PRESA	19.342	−96.42	42	AP6	45	36	8	0.7	M	NA	NA	NA	NA	NA	NA	NA	NA	NA	NA	NA	NA	NA	NA	NA	NA	3	NA
ANTIGUA_PRESA	19.342	−96.42	42	AP7	34	28	6	0.3	M	NA	NA	NA	NA	NA	NA	NA	NA	NA	NA	NA	NA	NA	NA	NA	NA	NA	NA
ANTIGUA_PRESA	19.342	−96.42	42	AP8	NA	NA	NA	0.6	M	2	NA	NA	NA	NA	NA	NA	NA	NA	NA	NA	NA	NA	NA	NA	NA	2	NA
ANTIGUA_PRESA	19.342	−96.42	42	AP9	41	34	6	0.5	M	NA	NA	NA	NA	NA	NA	NA	NA	NA	NA	NA	NA	NA	NA	NA	NA	1	NA
ANTIGUA_PRESA	19.342	−96.42	42	AP10	30	25	4	0.4	M	4	NA	NA	NA	NA	NA	NA	1	NA	NA	NA	NA	NA	NA	NA	NA	4	NA
ANTIGUA_PRESA	19.342	−96.42	42	AP11	43	35	10	0.7	M	3	NA	NA	NA	NA	NA	NA	1	NA	NA	NA	NA	NA	NA	NA	NA	NA	NA
ANTIGUA_PRESA	19.342	−96.42	42	AP12	33	25	5	0.4	F	NA	NA	NA	NA	NA	NA	NA	2	NA	NA	NA	NA	NA	NA	NA	NA	NA	NA
ANTIGUA_PRESA	19.342	−96.42	42	AP13	38	30	9	0.5	M	NA	NA	NA	NA	NA	NA	NA	NA	NA	NA	NA	NA	NA	NA	NA	NA	NA	NA
ANTIGUA_PRESA	19.342	−96.42	42	AP14	33	26	4	0.3	F	NA	NA	NA	NA	NA	NA	NA	3	NA	NA	NA	NA	NA	NA	NA	NA	1	NA
ANTIGUA_PRESA	19.342	−96.42	42	AP15	30	26	4	0.3	F	NA	NA	NA	NA	NA	NA	NA	NA	NA	NA	NA	NA	NA	NA	NA	NA	NA	NA
ANTIGUA_PRESA	19.342	−96.42	42	AP16	40	34	6	0.5	M	NA	NA	NA	NA	NA	NA	NA	NA	NA	NA	NA	NA	NA	NA	NA	NA	NA	NA
ANTIGUA_PRESA	19.342	−96.42	42	AP17	33	26	6	0.3	F	NA	NA	NA	NA	NA	NA	NA	NA	NA	NA	NA	NA	NA	NA	NA	NA	1	NA
ANTIGUA_PRESA	19.342	−96.42	42	AP18	33	24	7	0.3	M	NA	NA	NA	NA	NA	NA	NA	NA	NA	NA	NA	NA	NA	NA	NA	NA	NA	NA
ANTIGUA_PRESA	19.342	−96.42	42	AP19	36	29	7	0.5	F	NA	NA	NA	1	NA	NA	NA	NA	NA	NA	NA	NA	NA	NA	NA	NA	NA	NA
ANTIGUA_PRESA	19.342	−96.42	42	AP20	32	25	6	0.3	M	NA	NA	NA	NA	NA	NA	NA	5	NA	NA	NA	NA	NA	NA	NA	NA	NA	NA

## Experimental design, materials and methods

The study was conducted at 11 sites located between 42 and 1245 m above sea level (a.s.l.) within the La Antigua River basin. The La Antigua River is a high-gradient foothill river originating from the Cofre de Perote volcano and adjacent mountains from the Sierra Madre Oriental (altitude 4200 m) in the states of Puebla and Veracruz, Mexico. The river runs approximately 100 km east of the Gulf of Mexico [3]. We examined 19 *P bimaculatus* from a locality named Agua Bendita, 21 from another, Puente Nacional, and 20 from each of the other nine locations sampled in June 2016. Specimens were collected under collecting permit FAUT-0105. Fish were collected using DC backpack electroshockers, seines, and gill nets. Captured individuals were placed in plastic bags filled with water, transferred to the laboratory, and kept alive in aerated containers until subsequent examination for the presence of helminth parasites (within 8 h of capture). To complete the examination, fish were euthanised with an overdose of the anaesthetic 2-phenoxyethanol (Sigma-Aldrich, St. Louis, Missouri), protocol for the use of fish in research based on the NORM – 019 – STPS – 1993 established by the Instituto de Ecología, Pesquerías y Oceanografía del Golfo de México EPOMEX, Campeche, Mexico; specimens collected under the Cartilla Nacional de Colector Científico FAUT-0105 issued by the Secretaría del Medio Ambiente y Recursos Naturales [SEMARNAT] to GSM. Each fish was measured (total and standard lengths), and examined under a stereomicroscope in Petri dishes containing river water. Externally, the skin, scales, mouth, branchial cavity, anus, and fins of each host were examined. The branchial arches were removed, separated from the branchial cavity, and evaluated individually. All internal tissues, including the digestive tract, body musculature, and organs were examined for helminth parasites under a stereomicroscope in Petri dishes containing saline 0.7% solution. The helminths that were obtained from the dissections were counted and recorded separately for each fish. Internal examination included the inspection of all tissues and organs, viscera and muscles, except blood and bones, of each sampled fish. All helminths found except the Gyrodactylid monogeneans (see below) were isolated and counted, and then fixed in 4% hot formaldehyde (cestodes,monogeneans and adult digeneans, as well as larvae of digeneans and nematodes). Some monogeneans were fixed with ammonium picrate [1] and mounted unstained in gray–Wess medium [4], for analysis of sclerotized structures. Platyhelminths, including digeneans, monogeneans, and cestodes used for morphological examination of whole mounts, were stained with stained with either Mayer's paracarmine or Gomori´s triple stain dehydrated using a graded alcohol series, cleared in methyl salicylate, and mounted whole on Canada balsam. Nematodes were cleared in glycerine for light microscopy and stored in 70% ethanol. The Gyrodactylid monogeneans found were removed with the use of surgical needles and were preserved in 95% ethanol in Eppendorf tubes, and processed individually. Attachment organs (haptors) were excised under the dissection microscope and partially digested with a proteolytic solution to remove tissue enclosing the haptoral armature. Digestion was arrested by the addition of a 50:50 glycerine/formalin solution, and specimens were then coverslipped and sealed with nail varnish. Individual worm bodies that had their haptors excised were fixed in 95% ethanol and stored at −20 °C for further molecular analyses. Bodies of excised specimens whose haptors had been morphometrically characterized were placed individually in 1.5 ml Eppendorf tubes for genomic DNA extraction using DNeasy Blood & Tissue Kit (Qiagen, Valencia, California) following the manufacturer's instructions. The ribosomal region spanning the 3′ end of the 18S rRNA gene, ITS1, 5.8S rRNA gene, ITS2, and 5′ end of 28S rRNA gene was amplified by PCR (see [2]).

Taxonomic identification of helminths was performed based on morphometric analysis of the specimens, and in the case of Gyrodactylids monogeneans verified by molecular tools as explained above.

## Ethics statement

Protocol for the use of fish in research based on the NORM – 019 – STPS – 1993 established by the Instituto de Ecología, Pesquerías y Oceanografía del Golfo de México EPOMEX, Campeche, Mexico; specimens collected under the Cartilla Nacional de Colector Científico FAUT-0105 issued by the Secretaría del Medio Ambiente y Recursos Naturales [SEMARNAT] to GSM.

## Declaration of Competing Interest

The authors declare that they have no known competing financial interests or personal relationships that could have appeared to influence the work reported in this paper.

## Appendix Supplementary materials

Image, application 1
